# Towards a Better Understanding of the Potential Benefits of Seaweed Based Biostimulants in *Vitis vinifera* L. Cultivars

**DOI:** 10.3390/plants11030348

**Published:** 2022-01-27

**Authors:** Liam Jay Samuels, Mathabatha Evodia Setati, Erna Hailey Blancquaert

**Affiliations:** Department of Viticulture and Oenology, South African Grape and Wine Research Institute (SAGWRI), Stellenbosch University, Private Bag X1, Matieland, Stellenbosch 7602, South Africa; ljs@sun.ac.za (L.J.S.); setati@sun.ac.za (M.E.S.)

**Keywords:** climate, grapevine, biostimulants, seaweed extracts

## Abstract

Globally, 7.4 million hectares of arable land is planted with grapevine with a farm gate value of $68.3 billion. The production of grapes faces growing pressure associated with challenges such as climate change, diminishing resources as well as the overuse of chemical fertilizers and synthetic pesticides, which have an impact on sustainability. Consequently, viticulture has over the years embraced and implemented various practices such integrated pest management, organic and biodynamic farming to curb the high chemical inputs typically used in conventional farming. Biostimulants and biofertilizers are considered environmentally friendly and cost-effective alternatives to synthetic fertilizers and plant growth regulators. Seaweed is of particular interest because of its availability globally. It was reported that brown seaweed (*Ascophyllum* spp.) improves plant growth and agricultural productivity, hormonal signalling, and an improved secondary plant metabolism. It also provides an alternative to soil supplementation, avoiding some of the negative effects of fertilizers through the leaching of nutrients into groundwater sources. This review aims to provide a summary of the use of seaweed extracts in grape production and their influence on grapevine physiology and stress adaptation mechanisms.

## 1. Introduction

Grapevine functioning, development and production face growing pressure associated with abiotic (drought, salt, mineral nutrition disturbances, light and temperature) and biotic (wounding, pathogens, and herbivores) stressors [1]. The abovementioned stressors contribute to the overuse of chemical fertilizers and synthetic pesticides, which have an impact on agricultural sustainability [2]. Additionally, climate change poses a serious risk to job security, especially since the wine industry employs many low-skilled laborers [2,3]. Consequently, viticulture has over the years embraced and implemented various practices such as integrated pest management (IPM), organic and biodynamic farming to curb the high chemical inputs typically used in conventional farming [4]. Various farming strategies and practices have also been shown to influence grapevine microbiome structure and diversity [5,6,7]. Furthermore, the microbial phyllosphere and rhizosphere are negatively affected by the use of chemical products in associated vineyard environments [5].

Biostimulants and biofertilizers are considered environmentally friendly and cost-effective alternatives to synthetic fertilizers, plant growth regulators and crop improvement products [8]. A biostimulant is a substance, organism or a by-product of organic origin that is applied to a plant/crop to enhance performance in quality, yield, stress adaptation, and plant protective qualities [9]. Biofertilisers are products containing living microorganisms or natural substances that are able to improve soil chemical and biological properties, stimulating plant growth, and restoring soil fertility [9]. Due to differences in origin and function, biostimulants have been broadly classified into various categories [9]. Seaweed extracts are classified as biostimulants as opposed to bio-fertilizers as they “stimulate” internal responses (plant defense and growth) when applied to the plant, rather than providing plant-usable or plant-degradable supplementation [4]. For many years seaweed products have been utilized as animal feed, but their full agricultural potential has only recently been recognized [10]. Their use in eco-fertilizers and, recently, their incorporation in soil and foliar sprays in grape production, have proven to enhance numerous plant growth compounds [11]. Research has been directed at the study of seaweed extracts as biostimulants due to the complexity of their constituents [4]. Seaweed extracts are known to contain various forms of amino acids, carbohydrates, proteins, osmo-protectants and phytohormones [11]. Producers in the table grape industry have relied heavily on plant growth regulators (growth hormones) to control fruit set, enhance berry growth and size, and to overcome inadequate winter chilling [12]. The production of wine grapes seldomly uses growth regulators and plant hormones [12]. Interestingly, seaweed extracts have continually been associated with the hormone-like responses shown through improved yields as well as an increase in plant stress tolerance. The use of seaweed extract innovatively offers a promising sustainable approach to viticulture that is easy to incorporate into existing systems.

As biostimulants continue to gain support in the industry as an effective, sustainable alternative, most research is now aimed at understanding their mode of action and how they bring about their positive attributes on overall crop productivity. The effects on plant microbial eco-systems in grape production and functioning is also a topic that is gaining research popularity and is not yet fully explored and understood. Therefore, the demand for greener alternatives in grape production is growing amidst climate change. This review aims to discuss the contribution of seaweed extracts to mitigate abiotic and biotic stress on *Vitis vinifera* L. cultivars and their potential influence on grape and wine quality.

## 2. Biostimulants in Grapevine Cultivation: Classification

### 2.1. Biostimulant Definition

Plant biostimulants are defined as any substance or microorganism, of natural origin, applied to a plant with the aim of enhancing or improving nutrient efficiency, abiotic stress tolerance and crop quality traits, regardless of the nutrient content of the substance [9]. The term ‘biostimulants’ has been claimed by horticulturalists, referring to substances that promote plant growth without being nutrients, fertilizers, or pesticides. Nevertheless, plant biostimulants are more comparable to fertilizers, even though they do not act by means of supplementation [13]. Biostimulants are not intended to be applied for pathogen or pest protection, even if they promote defense responses within the plant. However, due to limited empirical data on the use of biostimulants, it is recommended that they be used in conjunction with existing conventional methods and not as the sole replacement for plant enhancement [14].

### 2.2. Biostimulants Classes

**Figure 1:**
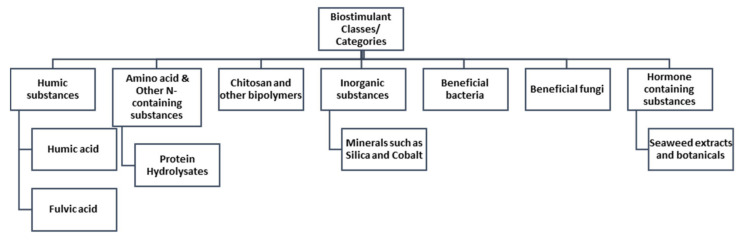
Different classes of biostimulants adapted from [9].

Biostimulants are broadly classified into seven groups which are widely recognized: (i) humic substances (HS), (ii) amino-acid and other nitrogen containing substances (AACP), (iii) chitosan and other biopolymers, (iv) inorganic substances, (v) beneficial fungi, (vi) beneficial bacteria and (vii) seaweed extracts and botanicals (HCP) (Figure 1) [9].

HS are natural components of the soil organic matter, resulting from the interaction between organic matter, the decomposition of plant, animal and microbial residues, and microbes in the soil and plant roots [9]. HS have been reported to stimulate plant root growth and development by expressing hormone-like activity and H+ ATPase activity in the plasma membrane [15].

**Figure 2:**
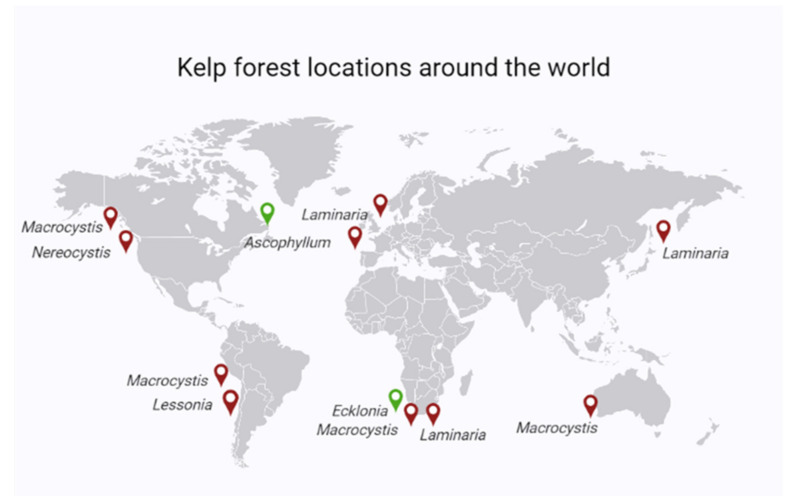
Location of the different kelp species forests around the world adapted from [19].

AACP, commonly referred to as protein hydrolysates (PHs), are mixtures of polypeptides, oligopeptides, and amino acids, obtained by chemical and enzymatic protein hydrolysis from both plant sources (crop residues) and animal wastes (e.g., collagen, epithelial tissues) [9]. Application of the products may be in the form of root applications or as foliar sprays. HCPs, such as seaweed extracts, contain identifiable amounts of active plant growth substances such as auxins, cytokinins, or their derivatives [9]. Seaweed extracts are described as one of the main groups of biostimulants with huge agricultural potential [16]. They are said to account for more than 30% of the global biostimulant market [17]. While there are three groups of seaweed (*Phaeophyceae*—brown*; Rhodophyta*—red and *Chlorophyta*—green) (Table 1), the brown species, *Ascophyllum nodosum* (AN) and *Ecklonia maxima* (EM) are mostly used for commercial production of seaweed extract [17]. Extracts have been explored for their ability to improve plant growth and agricultural productivity and have shown great potential for agricultural applications (Figure 2) [18].

## 3. Methods of Extraction: Possible Sources of Variation in Seaweed Biostimulants

The standing of seaweed extracts has been under great scrutiny over recent years, due to the inconsistent nutritional value of products and batches [20,21]. The manufacturing or extraction process of seaweed extracts proves to be the most challenging step in ensuring consistency and efficiency for its products [17,21,22]. It is believed that discrepancies in compositions can arise from the following sources: extraction process, origin (ecosystem) and anatomical variation [5,21,22]. In addition, product refinement/enrichment through the addition of nitrogen (N), phosphorus (P) and potassium (K) and/or preservatives can contribute to nutritional variation [21]. Significantly higher K and N levels were observed in Afrikelp^®^ and Basfoliar^®^ when compared to Kelpak^®^ [21]. Consequently, careful consideration should be taken when deciding on which commercial product to use.

The extraction of seaweeds can be achieved through physical and/or chemical methods, which include the use of variables such as heat and pressure as well as solvents [4,21,23]. The extraction process has often been used by producers as a competitive advantage for biostimulant production, and has thus been subject to professional secrecy [17]. These methods include water-based extractions, acid hydrolysis, alkaline hydrolysis, microwave-assisted extraction, ultrasound-assisted extraction, enzyme-assisted extraction, super critical fluid extraction and pressurized liquid extraction [21,23,24,25].

The most frequently used extraction process for commercial seaweed extract production entails an alkaline and acid hydrolysis, at high pressure [21,25,26]. This method has been proven to be the most successful, and consistent because of the high level of extractability and the moderate degradation of polysaccharides into oligomers which are one of the most biologically active components of seaweed extracts [21,26].

## 4. Grapevine Responses to Seaweed Application

Grapes are the world’s third most valuable horticultural crop [27]. These grapes can be used for: (i) wine production (57%), (ii) table grapes (36%) and (iii) dried grapes (raisins) (7%). Grapevine establishment, growth, physiological and metabolic processes, and yield are dependent on the health status and the nutritional status of the soil and the plant throughout the growing season. The role of seaweed in grapevine is poorly understood and will be discussed.

### 4.1. Seaweed Extract Effect on Grapevine Physiology

**Figure 3:**
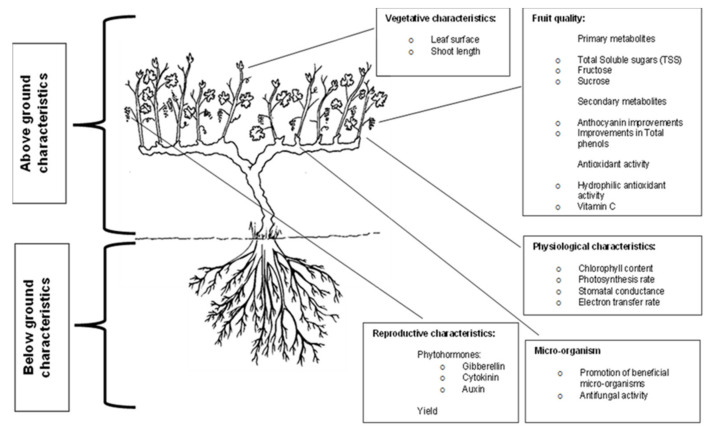
Benefits associated with the use of an *Ascophyllum nodosum* based seaweed extract biostimulants adapted from [17].

Seaweed extracts have been known to improve plant physiology at establishment in the nursery and when planted in a commercial vineyard to ensure a well-established root architecture [28]. Many studies have proved that seaweed extracts possess characteristic growth stimulating properties as they alter physical, biochemical and biological properties of the soil and may also affect the architecture of plant roots facilitating for the successful uptake of water and minerals (Figure 3) [9,12,21,29,30,31,32]. They have the ability to “speed-up” the secondary metabolic pathways within the plant by triggering internal mechanisms [14]. Turan and Köse (2004) found that seaweed extract was more effective in supporting Cu uptake in vines [33]. Sabir et al. (2015) reported that seaweed enhanced the leaf Zn chlorophyll content of the grapes [32]. Irani et al. (2021) found that seaweed treated grapevines had higher concentrations of N, P, K, Fe, and Zn than untreated vines under well-watered conditions [33].

Frioni et al. (2018) reported that AN applications had minor effects on vine physiological performances as related to carbon assimilation and vegetative growth [34]. Furthermore, AN extract treatment had no effects on leaf gas exchanges and supports the findings of other authors suggesting that seaweed-based extracts work more effectively if stress is induced [29]. Salvi et al. (2019) reported that foliar treatments with AN increased photosynthesis and stomatal conductance in treated compared to control plants [35]. Moreover, grapevines treated with seaweed were able to maintain the potential efficiency of Photosystem II close to the optimal value during the hottest periods. Stem water potential was not impacted using AN extract [26,36,37]. The enhancement of physiological performances promoted by an application may be related to the presence of amino acids and phenolics, which probably confers high DPPH Radical-Scavenging Capacity to the extract. Numerous studies suggested that protection against extreme osmotic stress is provided by drought tolerance [25,36,37]. Proline acts as a cytoplasmic osmolyte and as a ROS-scavenging compound and thus is poorly involved in leaf osmotic adjustments [37].

Tombesi et al. (2021) studied the impact of extract impact on grapevine gas exchange under well-watered (field capacity throughout the experiment ~6 L per vine per day and water stress conditions and to examine its mode of action under stress (light and temperature) [38]. The application of AN caused a slight increase in stomatal conductance that resulted in an increase in water plant conductivity to atmosphere. Increased transpiration induced by AN improved leaf thermoregulation, facilitating vine recovery after a stress period. The latter can be seen as a double-edged sword as it can result in faster water resource depletion and higher vulnerability of the xylem. AN increased transpiration through a reduction of stomatal sensitivity to Vapor Pressure Deficit (VPD) [38].

### 4.2. Seaweed Extract Effect on Fruit Quality

Fruit quality is essential to ensure the production of good wine. The parameters which determines the quality of the fruit include primary (Total Soluble Solids—TSS, pH and Titratable Acidity—TA) and secondary (phenolic compound) metabolites as well as yield components (bunch weight and berry weight) [9]. The tempo of accumulation of the primary and secondary metabolites are greatly influenced by the abiotic factors [39,40,41]. Primary metabolites include organic acids, proteins, nucleic acids, etc., while examples of secondary metabolites include grape phenolics which are broadly divided in to two main groups, namely flavonoids and non-flavonoids. The three main groups of flavonoids identified in red grape berries are flavan-3-ols (tannin), anthocyanin, and flavonols [42]. Seaweed extracts contain an array of secondary metabolites, which are potentially the key behind their growth response characteristics [4,38]. Frioni et al. (2018) reported a slightly positive TSS evolution in Pinot noir during the first part of the ripening process treated with Acadian Marine Plant Extract Powder extracted by alkaline hydrolysis [35]. Irani et al. (2021) found that drought-stressed berries had significantly higher TSS and TA content. Under drought conditions, an application of seaweed extract significantly enhanced the weight of the berries, improved yield, and TSS, and decreased TA [33]. The manufacturer and the extraction process is unknown for the study in [33].

Deng et al. (2019) reported that SUNRED© (proprietary mixture containing 26.6 g·L^−1^ of organic N, 13.3 g·L^−1^ of mineral N, 93.1 g·L^−1^ of K_2_O, and 186.2 g·L^−1^ of organic C) a biostimulant, whose main component is seaweed extract, increased the phenolic attributes (i.e., anthocyanin content in the skins) through upregulating genes involved in the anthocyanin biosynthesis pathway [43].

Frioni et al. (2021) also investigated the effects of a seaweed extract and observed similar positive results of Acadian Marine Plant Extract Powder© on anthocyanin, and phenolic concentration in grapes [44]. Induced drought-stress significantly reduced the berry weight of (*Vitis vinifera* L.) cv. Yaghoti in comparison to well-watered vines [33]. Grape yield, cluster and berry size were not impacted by AN extract applications but accelerated véraison improved anthocyanins accumulation in all cultivars and increased phenolic content, particularly in Sangiovese [29]. Salvi et al. (2019) reported that AN treated Sangiovese grapevines allowed increasing the number of berries and anthocyanin extractability in two consecutive seasons [35].

Salvi et al. (2019) reported that an increase in temperature by 1 °C resulted in delayed ripening of AN treated Sangiovese grapevines treated with Acadian Marine Plant Extract Powder extracted by alkaline hydrolysis [35]. This resulted in grapes with lower sugar, but higher anthocyanins. Despite having positive berry quality traits no yield increases were found. This can be because of the late application on fully developed berries and cluster. Numerous authors reported that the application of biostimulants during stage I of berry development (cell division and enlargement) could induce an improvement in berry weight and yield in table grapes [45,46].

Taskos et al. (2019) study also showed an increase in yield by 25–36% in treated Merlot vines when using fresh algae which was washed, shredded, and added to water. The latter solution was acidified with concentrated 95% sulfuric acid to pH 3 [12]. Grapevine inflorescence primordia are initiated in the summer of the year preceding to that in which they flower. Numerous factors influence grapevine fertility (intrinsic factors—genetics, morphology, physiology, carbohydrate reserves, nutrient reserves, plant hormones; and extrinsic factors—light, temperature, water; and viticultural practices—canopy management and pruning). It has been reported that seaweed extracts can also be used to trigger berry fruit set in grapevine [47,48]. As fruit set and the number of flowers increase, so does the corresponding yields. Often these responses correspond to the phytohormone levels present in the extracts [5]. Anlagen formation is influenced by the hormone levels found in the grapevine as well as exogenous application thereof. Gibberelin and cytokinin are the two main regulators involved in flowering. Gibberelin is applied before the initiation process, it will result in an inhibiting effect on flower bud formation. However, numerous authors reported that cytokinin has an influence on fertility during formation of inflorescence primordia and formation of flowers [49,50,51]. A recent study conducted by Ali (2019) showed that plants treated with seaweed extracts display a triggered gene response to produce endogenous phytohormones [52]. In terms of yield components, Arioli (2021) reported an increase in grape yield (of up to 14.7%) through fertigated soil applications of seaweed extract of the two large cold-water species: *Durvillaea potatorum* (native to the southern hemisphere) and AN (native to the northern hemisphere), in five geographically different Australian locations, spanning across five seasons (2012–2017) [53]. The cultivars investigated were Chardonnay, Semillon, Merlot and Cabernet Sauvignon. It was possible to monitor the impact of seaweed extract on yield in the latter study as the study was conducted over a period of five seasons.

### 4.3. Effect of Seaweed Extract on Grapevine Phytohormone Levels

The similarity between the growth responses demonstrated and the responses seen after phytohormone applications, has added to the speculation that these phytohormones are present in seaweed extracts [11]. Because of the diversity of the plant responses to the applications of seaweed extracts, the possibility that more than one group of hormones may be present is highly likely. Some of the phytohormones isolated from brown seaweed extracts are: cytokinin, auxin, gibberellin, ABA, indole-3-acetic acid, ethylene, brassinosteroids, jasmonates, salicylic acid, strigolactones, zeatin, kinetin and BAP (cytokinin-6-benzylaminopurine) [5,21,48]. Despite all these phytohormones being extracted from the seaweed there are several critical questions which need to be answered to elucidate the research to real-world applications of seaweed extracts (i.e., rate of application; mode of application; time intervals of application; extraction method of the product; environmental conditions during application; and mode of action) to fully understand the contribution of seaweed extract in grapevine functioning.

Most application types are either foliar, root application or a combination of both [4]. Seaweed extracts can also be added through fertigation, dipping and drenching [4,52,53]. The frequency and time intervals of seaweed application (phenological stage) was achieved at véraison and throughout maturation in most studies [34,35,52,53,54,55]. Taskos et al. (2019) applied seaweed extract from fruit set and saw a 26% increase in yield. From this it can be deducted that an impact on yield will only be seen if the product is applied at fruit set when cell division and enlargement takes place [12]. If the product is applied at véraison and thereafter, the secondary metabolism is targeted which will result in an increased phenolics [29,56]. Arioli et al. (2021) studied seaweed extract applications to soil in five locations, across three Australian states and four cultivars over a period of five years. The reoccurring application of seaweed extracts (EL stage 4 until EL stage 34) proved that seaweed extract applications is an economical option for sustainable viticulture [53]. Foliar applications prove to have a better result as compared to the other forms of application due to the immediate contact of the product by the leaves. This warrants an almost immediate uptake of the product by the plant while a root application will first be absorbed by the soil particles which will result in a reduction of the mobility. These products are then applied with a sprayer (handheld/gasoline backpack sprayer). The optimal application times for these extracts were determined to be around every 10–14 days for provoking the best plant responses [53]. It has been showed that biostimulants functions optimally if a stress scenario already exists in the plant [33,38,53]. It is also advised by seaweed producers that the application of the product occurs at low physiological activity (early morning 05:00–07:00) [56].

## 5. The Role of Plant Hormones and Grape Berry Development and Ripening

**Figure 4:**
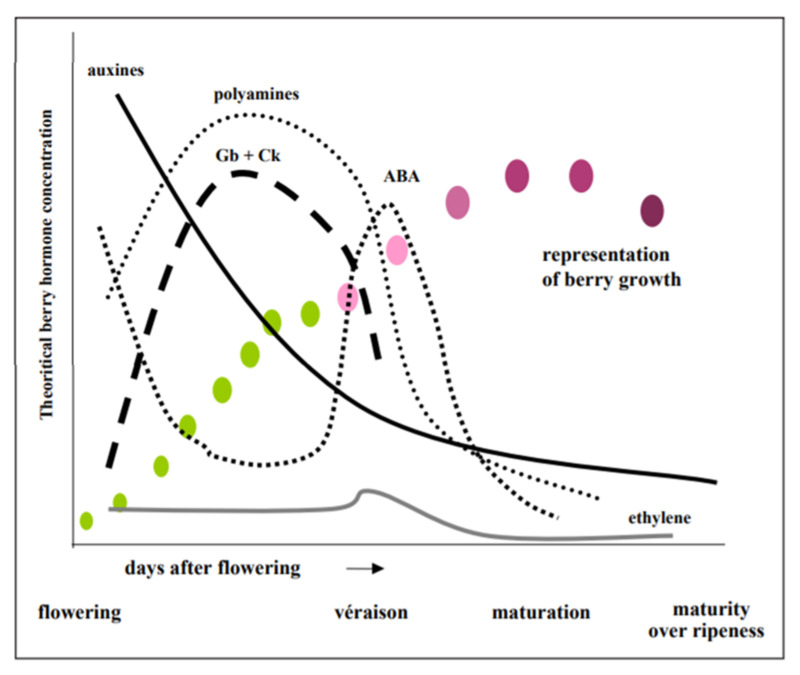
Theoretical evolution of plant hormones during berry development. Gb: gibberellines; Ck: cytokinins; ABA: abscissic acid [57].

Grape berry development involves a complex series of physical and biochemical changes. These can be divided into three major phases: (i) green growth, (ii) the lag phase and (iii) ripening phase [42]. Plant hormones occur naturally at low concentrations in vegetative tissues of plants and can also be added endogenously (Figure 4) [57]. The endogenous application of the plant hormones can be mostly achieved through the addition of products like seaweed extracts. The changes in berry tissues are usually associated with response due plant hormone regulation in the berry. The levels of these hormones fluctuate, depending on the phenological stage of development and exist in low concentrations (10^−9^ and 10^−5^ M [57]. The most important hormones that play a role in berry ripening and maturation are auxins, cytokinins, giberillins, abscisic acid and ethylene [57]. Auxins and cytokinins exist at a maximum concentration in the berry pulp before reaching the green growth stage, after which they decrease (Figure 4). This drastic decrease usually happens around the time of véraison [57]. Abscisic acid (ABA) alternatively, responds oppositely to auxin and cytokinin, and reaches maximum levels at the herbaceous plateau, then rapidly decreases at the onset of maturation [57].

## 6. Increased Tolerance to Environmental Factors: Abiotic Stress Tolerance

Various environmental factors, such as extreme temperatures, light intensities, soil salinity and frost are the realities of climate change and can be devastating to viticulture [1]. Abiotic stress is often linked to physiological responses by the plant which can include the synthesis of plant protective compounds [58]. Through the continual exposure to such abiotic stress, an accumulation of the reactive oxygen species (ROS) including free radical and nonradical molecules, will cause irreversible damage to the plant [59,60]. Certainly, various environmental stressors trigger the production of a vast array of compounds and activators which all play a role in carbon, nitrogen, sulfur and minerals’ metabolism [58].

The modes of action of seaweed extracts have been categorized into three main groups, namely: (i) improved nutrient acquisition (improved growth and vigour), (ii) inhibition of chlorophyll degradation (improved tolerance to abiotic stress) and (iii) defence response triggers to biotic stressors (improved tolerance to biotic stress) [5].

(i) Improved nutrient acquisition

Seaweed application triggers hormone biosynthesis which results in improved nutrient acquisition in tomato and bell pepper [4]. The suggested mode of action is an upregulation of the genes involved in auxin, gibberellin and cytokinin production. In instances of prolonged periods of water-stress, plants often respond through a decrease in the uptake of mineral elements from the soil. In a study done by Irani et al. (2021) it was found that in drought-stress conditions, the concentrations of the macro-elements N, P, and K in the leaves increased, and the concentrations of the micro-elements Fe and Zn decreased [33]. However, when these drought-stressed vines were treated with a foliar application of seaweed extract, a higher level of these nutrients were obtained as opposed to the control vines.

(ii) Inhibition of chlorophyll degradation (drought, salinity, and cold tolerance)

Betaines have been found to be responsible for the inhibition of chlorophyll degradation in plants [61]. It has been reported that glycine betaine, a component of seaweed extract, protects cells from various abiotic stressors pertaining to osmotic balance such as that experienced with salinity stress in plants. It achieves this through the protection of complex proteins, antioxidant enzymes and the Photosystem II complex [62,63]. The physiology of potted Cabernet franc grapevines was not impacted when AN extract was applied [34]. In a study performed by Irani et al., (2021) although drought stress resulted in chlorophyll degradation, seaweed extract applications alleviated the negative effects of drought on chlorophyll content [33].

Shukla et al. (2018) reported an improved drought tolerance in *Arabipdopsis* after being treated with AN extracts [64]. It was further discovered that the upregulation of the genes involved in ABA catabolism as well as the detoxification of ROS (GmGST, GmBIP and GmTP55) were involved [64]. Shukla et al. (2019) reported that seaweed extracts could promote the up- and down-regulation of many genes, which is dependant and the extract manufacturing process and pH of the seaweed extract itself [64]. Alkaline extracts reduce gaseous exchange, transpiration rate, and promote ABA synthesis, whereas acidic extracts cause the opposite metabolisms [65]. Salinity stress tolerance was significantly improved when *Arabidopsis* was treated with a seaweed extract [66]. This was attributed to the upregulation of 257 genes in salt-induced stress: (late embryogenesis abundant 3 family and transcription factor circadian clock) [67].

The response of the plants subjected to abiotic stress in combination with seaweed extract treatment resulted in improved physical characteristics of the root morphology, accumulation of non-structural carbohydrates which improve the storage of energy, enhanced metabolism and water adjustments, as well as the build-up of proline [66].

(iii) Defence response triggers by biotic stressors

The cell walls of the seaweed extracts are made up of polysaccharides, such as ulvans, laminarins and carrageenans, which are associated with some resistance in plants [66]. The bioactive polysaccharides are known to trigger defence responses via salycic acid, jasmonic acid and ethylene [4]. As this reactions take place, various proteins (such as pathogenesis-related proteins), enzymes (such as chitinases and glucanases) and phenolic molecules accumulate, which act as security against many pathogens [4]. Though there are few reports on the use of seaweed extract as a biocontrol, few studies demonstrate their potential benefits. These beneficial characteristics of seaweed extracts have been further attributed to the presence of pathogen-inhibitory polysaccharides in the composition of these products [9]. A field trial conducted on tomato and sweet pepper as model crops further demonstrated the bio-control abilities of *AN* sprays. Reduced infestations of *Xanthomonas campestris* was seen through the upregulation of genes involved in salicylic acid, jasmonic acid and ethylene synthesis as well as biosynthesis of the defence enzyme [9].

## 7. Prevention of Grapevine Pests and Diseases: Biotic Stress Tolerance

The pressures brought about through climate change and the overuse of chemical pesticides have increased the occurrence of resistant pathogens and pests, preying on weakened crops [4]. Interestingly, it has been noted that seaweed extracts also have the ability to induce plant defence responses against infection from bacteria, fungi and some viral pathogens [9,59]. Fungal pathogens, such as *Botrytis cinerea*, also known as “grey rot or grey mould”, *Plasmopara viticola* (downy mildew) and trunk diseases, such as *Eutypa lata,* are common problems experienced by grape producers in areas with high summer rainfall. With limits to the use of copper-based formulations in organic production systems, organic alternatives are constantly being welcomed. Notably, in a study performed by Aziz et al. (2003) seaweed extracts, derived from *Laminaria digitata*, showed fungicidal properties, significantly reducing the incidence of fruit rot by 55–75% [68]. When *Laminarin* was combined with copper at a low concentration in Montepulciano, downy mildew was significantly reduced on grape berries compared to a Bordeaux mixture of copper hydroxide, chitosan (99.9%), bentonite, vermicompost extract and untreated vines [69]. These properties have been attributed to many compounds located within their makeup, but mostly due to a group of polysaccharides present in most extracts that are able to chelate metal ions [14]. These polysaccharides include alginates, fucans, ulvans, laminarin (from brown algae). Compounds such as mannose and mannitol alcohols, responsible for osmotic process regulation, also cause plant pathogen inhibition [14]. The activation of defence responses in grapevine was seen when ulvans (a large polysaccharide component of seaweed extract products) were applied via a foliar application that saw induced protection against downy mildew and powdery mildew [66,70,71]. The ability of the plant to defend itself from attack, can be attributed to a general increase in plant vigour of the treated plants, which showed resistance induced systemic or systemic required resistance or enhanced soil suppressiveness as a result of a modified phyllosphere [4].

**Figure 5:**
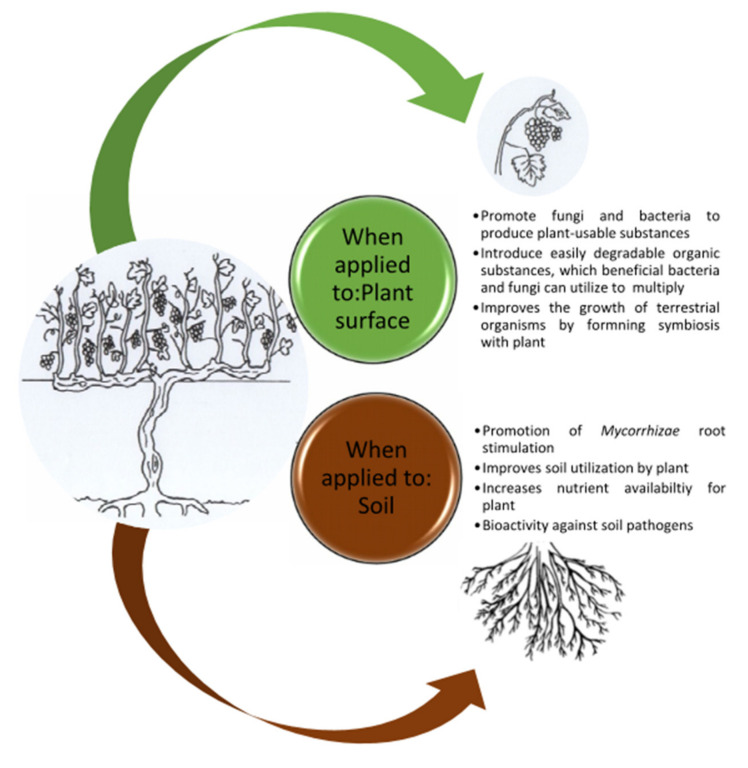
Rhizopshere benefits of the application of seaweed extract biostimulants.

Nematodes are known to cause severe damage to crops and have been identified as one of the organisms that have been proven to be successfully controlled using seaweed extract soil applications [52]. This nematocidal activity is, however, not directly related to seaweed nematocidal characteristics but has been largely attributed to the plants intrinsic self-defence responses triggered by seaweed applications [72]. In addition to nematode control, seaweed extracts were able to significantly reduce infestation caused by borers, aphids and thrips in sugarcane thus preventing great economic loss (Figure 5) [73,74].

## 8. Seaweed Extract Effect of Microbiome

The plant rhizosphere and phyllosphere includes all plant roots and leaves surfaces, respectively [4]. The interaction between all the microorganisms present on these surfaces have a major influence on plant growth and function. These plant surfaces (both internal and external) excrete several utilizable substances, which include sugars, and organic acids, which benefits microbial populations present on and in the plant [75]. As various chemicals and substances are applied, either via foliar spray or soil drench treatment, the excretory composition of the plant is subject to change [4]. The growth responses associated with the application of seaweed extracts has also been attributed to the rhizosphere manipulations brought about through their use [76].

Though the main purpose of agricultural practices, aids and management processes is to improve yield, this is often achieved at the expense of disturbing the microorganismal population (mycorrhiza fungi and beneficial organisms) on the plant surface [77]. Traditional pesticides, fertilizers and irrigation practices are all known to reduce microbial populations [78]. These negative effects can be reversed by the supplementation of more natural products, such as seaweed extract biostimulants, which promote rhizosphere development. Very little research has been conducted on the effects of biostimulants, on grapevine microbiome, let alone the impacts seaweed extracts have on grapevine microbiology [76]. Though grapevine microflora is not directly linked to improvements in yield, quality and grapevine physiology, their secondary effects and the symbiosis between microbe and vine, is what is believed to have drastic impacts on grapevine functioning and development [75,76].

The scarcity of research conducted on the impact of seaweed extract biostimulants on microbial communities sparked the interest for a study conducted by Renaut (2019) [76]. A commercially available *AN* extract’s effects on fungal and bacterial colonies in the soils of tomato and pepper plants were studied. Amplicon sequencing was used to monitor any shift in the microbial community during the experiment. The study concluded that AN extract application to soil positively influenced fruit, shoot and root activity significantly [76]. Similarly, a study conducted on maize using an extract, showed that the microbial phyllosphere was significantly altered when the extract was applied. Dominant microbes were different as well as species diversity in the treated plants [4]. There is a growing desire by researchers to fully understand the relationship between seaweed extract application and the microbial phyllosphere and rhizosphere. The use of metagenomics and amplicon sequencing provide valuable resources and tools to better justify the need for seaweed extract use in sustainable viticulture.

## 9. Effects on Winemaking

Yeast-Assimilable Nitrogen (YAN) levels in grape musts are important for ensuring a healthy fermentation. Grape musts with suboptimal YAN levels can often result in sluggish or stuck fermentations and the development of off flavours in the resulting wines [79]. Moreover, one of the many factors influencing the final quality of fermentation is determined by the nitrogen levels of the berries at harvest [80]. In Tempranillo blanco vines treated with an AN seaweed extract, levels of the amino acids, tryptophan and leucine were increased compared to a control [79,80,81]. The amino acid leucine is closely related to the production of isoamyl acetate and isoamyl alcohol, which is responsible for the banana and fruity aromas in wine [43,82]. 

Red wine quality is often determined by the phenolic content in the grape berries. When AN was applied Pinot noir and Cabernet franc grapevines increased anthocyanin and total phenolic content was seen in the harvested fruit [29,81]. Salvi et al. (2019) reported a big variation in the anthocyanin amount of Sangiovese grapes treated with seaweed extract [35]. The latter was ascribed to the higher radiation and temperature and lower rainfall in 2017 compared to 2016 [33]. Furthermore, the partitioning of secondary metabolites (i.e., anthocyanins and flavonols) were higher in the treated grapevines compared to the control grapevines. The ratio of methoxylated to non-methoxylated anthocyanins was lower in treated than in control grapevines vines. AN-treated grapevines showed hydroxycinnamic acids both in berry skins and in leaves and showed a reduction in the biosynthesis of methoxylated anthocyanins, which are usually accumulated in grapes under environmental constraints [42]. Garde-Cerdán et al. (2021) reported that delphinidin-3-glucoside, cyanidin-3-coumarylglucoside, malvidin-3-cumaroylglucoside and the acetylated anthocyanins including its total content, whose concentration was significantly higher in 2017 samples than in the 2018 ones [83].

From the abovementioned seaweed, applications to the grapevines differentially affected phenolic compounds in grapes depending on season. Seaweed can thus act as an elicitor in grapevines allowing the triggering of stilbene synthesis in the grape berries.

## 10. Conclusions

From the above, it is clear that a gap exists in the knowledge of the use of seaweed extract on the functioning of *Vitis vinifera* L. commercial cultivars. Seaweed extracts in viticulture can be seen as an underutilized eco-resource. Seaweed extracts improve the overall growth and functioning of grapevines, by increasing resistance to drought stress and susceptibility to fungal diseases and ensuring to improve crop quality. Improved growth associated with the application of seaweed extracts can also be attributed to the ability of these products to modify the microbiological environment (phyllosphere) of the grapevine. Despite the positive contributions of seaweed based biostimulants reported in literature ample questions remain: which seaweed product should be used? What is the application method (aerial or soil) and time of application (early/mid-morning/evening) (need for re-application)? What is the mode of action on the primary and secondary metabolites pathways, climatic conditions during application and at the location? Seaweed cannot be used solely, but in combination with current conventional products. Seaweed extracts can be an alternative and sustainable management tool. In order to achieve the latter, the application of research to real world producers will be advantageous to an agricultural sector of such importance.

## Figures and Tables

**Table 1:** Visual comparison of the colour of the three main groups of seaweed species adapated from [9,20].

Seaweed Species Groups:	
1. *Phaeophyceae* (brown) e.g., *Ecklonia cava*	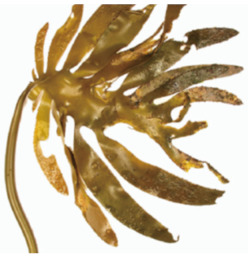
2. *Rhodophyta* (red) e.g., *Marginisporum aberrans*	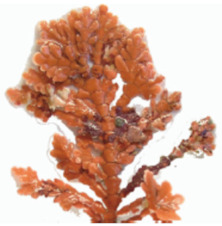
3. *Chlorophyta* (green) e.g., *Ulva pertusa*	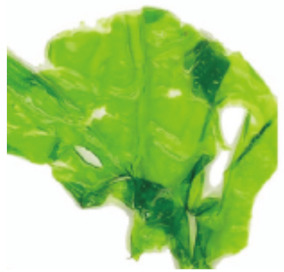

## Data Availability

No new data was created or analysed in this study.
